# Improved Rifamycin B Production by *Nocardia mediterranei* MTCC 14 under Solid-State Fermentation through Process Optimization

**DOI:** 10.1155/2014/621309

**Published:** 2014-10-09

**Authors:** Basavaraj M. Vastrad, Shivayogeshwar E. Neelagund, Sudhir R. Iiger, Ajeet M. Godbole, Venkatrao Kulkarni

**Affiliations:** ^1^Department of Pharmaceutical Biotechnology, S.E.T's College of Pharmacy, Dharwad, Karnataka 580002, India; ^2^Department of PG Studies in Biochemistry, Kuvempu University, Jnana Sahyadri campus, Shankaraghatta, Shimoga 577 451, India; ^3^Department of Pharmaceutics, S.E.T's College of Pharmacy, Dharwad, Karnataka 580002, India; ^4^Department of Pharmacology, S.E.T's College of Pharmacy, Dharwad, Karnataka 580002, India

## Abstract

Optimization of various production parameters using response surface methodology (RSM) was performed to assess maximum yield of rifamycin B from *Nocardia mediterranei* MTCC 14. Plackett-Burman design test was applied to determine the significant effects of various production parameters such as glucose, maltose, ribose, galactose, beef extract, peanut meal, ammonium chloride, ammonium sulphate, barbital, pH, and moisture content on production of rifamycin B. Among the eleven variables tested, galactose, ribose, glucose, and pH were found to have significant effect on rifamycin B production. Optimum levels of the significant variables were decided by using a central composite design. The most appropriate condition for production of rifamycin B was found to be a single step production at galactose (8% w/w), ribose (3% w/w), glucose (9% w/w), and pH (7.0). At these optimum production parameters, the maximum yield of rifamycin B obtained experimentally (9.87 g/kgds dry sunflower oil cake) was found to be very close to its predicted value of 10.35 g/kgds dry sunflower oil cake. The mathematical model developed was found to fit greatly with the experimental data of rifamycin B production.

## 1. Introduction

Rifamycin B is powerful, less toxic, and easily biodegradable antibacterial ansamycin antibiotic [1] and is produced by strain of* Nocardia mediterranei*. It bear distinct critical antibacterial activities [2], including tuberculosis, leprosy, and AIDS-related mycobacterial infections [3]. So rifamycin B has become one of the most effective antibacterial ansamycin antibiotics.

Processes that can be used for rifamycin B production include submerged fermentation (SmF) and solid-state fermentation (SSF). The solid-state fermentation is particularly beneficial for antibiotics production by filamentous actinomyces, since it simulates the natural habitat of these production strains [4, 5]. Another benefit of SSF is that agroindustrial residues (sesame oil cake, soybean cake, coconut oil cake, mustard oil cake, palm kernel cake, groundnut oil cake, cottonseed cake, canola oil cake, olive oil cake, rapeseed cake, etc.) can be used as the solid substrate, acting as sources of both carbon and energy. However, certain operational conditions of SSF, such as barrier in controlling. The moisture content of the substrate and avoiding heat buildup have bounded its industrial application. The influence of variety of nutrients, pH, and substrate moisture content on the kinetics of growth and rifamycin B formation is crucial for SSF process scale-up [6]. The strains that have been considered for the production of rifamycin B under SSF include* Amycolatopsis* sp. RSP 3 [6].

Oil cakes have chief nutritional value and are possibly valuable substrates for use in biotechnological SSF processes for the manufacture of chief value products such as antibiotics [7, 8] and enzymes [9, 10]. The development of new sunflower varieties has enabled edible oils to be obtained from rapeseed, which is now the world's second largest source of edible oil. The by-product of oil extraction, sunflower oil cake, not only is a rich source of nitrogen, carbon, and minerals but also is ample and economical [11]. However, despite the ability of* Nocardia* strains and their certified applications in antibiotics production, to the best of our apprehension there have been no studies concerning the characterization of these actinomyces for the production of rifamycin B using sunflower oil cake as solid substrate in SSF.

Plackett-Burman design and response surface methodology (RSM) are competent statistical techniques for optimizing complex processes because it allows additional, capable, and easier adaptation and explication of experiments compared to conventional methods [12, 13]. In addition, it is less arduous and time consuming than other conventional methods to optimize a process. It is extensively used for optimization of the process conditions of the fermentation medium [14–16].

In this paper, we describe the optimization of appropriate fermentation medium conditions for rifamycin B production, with the help of both conventional and statistical optimization techniques.

## 2. Materials and Methods

### 2.1. Microorganism, Preparation of Inoculum

*Nocardia mediterranei* MTCC 14 was obtained from the Microbial Type Culture Collection and Gene Bank (MTCC), Chandigarh, India, and was used in this study for rifamycin B production. The strain was maintained on agar-slant medium (pH 8.0) consisting of (g/L) dextrose 20, glycerol 20, yeast extract 5, beef extract 3, casein hydrolysate 3, peptone 2.5, malt extract 1, and agar 20 and stored at 48C. These slants were subcultured on regular intervals. The spores were harvested and suspended in sterile distilled water containing 0.01% (v/v) Tween 80 to obtain approximately 2.0 × 10^6^ spores/mL.

### 2.2. Raw Materials

Sunflower oil cake was collected fromRaja Fat and Feeds Private Limited, Rajpura, Punjab, India. The collected sunflower oil cake was first dried and mechanically milled with a lab mill (Ultra Centrifugal Mill) and sieved through standard mesh sieves (200–500 *μ*m) using an electronic sieve shaker to obtain the powder of 200–500 *μ*m particle sizes.

### 2.3. Optimization for Rifamycin B Production by One-Variable-at-a-Time Approach

SSF was carried out in a 250 mL Erlenmeyer conical flask containing 4 g solid substrate (sunflower oil cake). In the present paper, the requirement of medium components including various carbon (5% [w/w], glucose, maltose, galactose, ribose, and xylose) and nitrogen sources (7% [w/w], peanut meal, beef extract, soybean meal, ammonium sulphate, and ammonium chloride), barbital (1%, 2%, 3%, 4%, and 5%), pH (7.0, 7.5, 8.0, 8.5, and 9.0), and moisture content (40%, 45%, 50%, 55%, and 60%) were optimized. The sunflower oil cake was supplemented with (in mg) soybean meal 250, calcium carbonate 75, potassium nitrate 100, barbital 20, and magnesium sulphate 1 with predetermined quantity of water. The contents of the flasks were mixed thoroughly and autoclaved at 121°C for 15 min at 1 kgf/cm^2^. After cooling the flasks to room temperature, the flasks were inoculated with 2.0 × 10^6^ spores/mL under sterile conditions [6]. The pH adjustment of solid medium was achieved by adjusting the pH of moisturizing medium before being added to the solid material. The contents of the flasks were fully mixed and incubated at 28 ± 0.5°C for predetermined time period and used for extraction and estimation of rifamycin B.

### 2.4. Extraction of Antibiotics

Enzyme extraction was carried out by mixing fermented mass with distilled water for 1 h on an orbital shaker at 200 rpm. Contents of the flasks were filtered through muslin cloth and the filtrate was centrifuged at 2070 ×g for 10 min. The supernatant obtained was used for rifamycin B estimation.

### 2.5. Estimation of Rifamycin B

Rifamycin B was estimated according to the method of Pasqualucci et al. [17]. Reaction mixture contained 0.1 mL of suitably diluted antibiotics + 5 mL of 0.2 M acetate buffer (pH 4.63) and the absorbance was read at 425 nm using UV-Visible spectrophotometer (SHIMADZU UV-1800), against 0.1 mL of antibiotic solution diluted to 5 mL with acetate buffer, pH 4.63, containing 0.1% NaNO_2_. Rifamycin B production under SSF was expressed as g/kgds dry fermented mass. Each sample was tested in duplicate.

### 2.6. Experimental Design

Single factor experiment was used to select the best carbon and nitrogen sources, barbital, pH, and moisture levels.

### 2.7. Response Surface Methodology

Response surface methodology was applied in two stages, first to identify the significant processes parameters for production of rifamycin B using Plackett-Burman design test and later the significant processes parameters which resulted from Plackett-Burman design were optimized by using a central composite design. The experimental design and statistical analysis of the data were accomplished by using Stat-Ease Design Expert statistical software package (trial version7).

### 2.8. Plackett-Burman Design

Plackett-Burman design test was applied to describe the significant variables responsible for production of rifamycin B from* Nocardia mediterranei* MTCC 14. This design test assumes that there are no interactions between the different production parameters and is based on the first-order model:

(1) $$\begin{array}{c} Y=\beta_{0}+\sum \beta_{i}x_{i}, \end{array}$$

where *Y* is the estimated direct function, *β*_0_ is the scaling constant, *β*_*i*_ is the regression coefficients, and *x*_*i*_ is the variables. The effect of eleven variables with coded notation (glucose (*X*1), maltose (*X*2), ribose (*X*3), galactose (*X*4), beef extract (*X*5), peanut meal (*X*6), ammonium chloride (*X*7), ammonium sulphate (*X*8), barbital (*X*9), pH (*X*10), and moisture content (*X*11)) on the production of rifamycin B was tested at two experimental levels, high level denoted by (+) and a low level denoted by (−), as listed in Table 1. Eleven variables were screened by conducting 12 experiments and the experimental design is given in Table 2. All experiments were conducted in duplicate and the average value of extracted rifamycin B was used for statistical analysis.

The variables which were significant at 5% level (*P* < 0.05) from the regression analysis as given in Table 3 were reasoned to have major effect on production of rifamycin B and were further optimized by central composite design.

### 2.9. Central Composite Design

A central composite design was applied to determine the optimum level of four significant production parameters screened from Placket-Burman design test. As shown in Table 4 the effect of four processes parameters (galactose (*X*4), ribose (*X*3), glucose (*X*1), and pH (*X*10)) on the production of rifamycin B was calculated at five experimental levels, −*α*, −1, 0, +1, and +*α*, where *α* = 2^*n*/4^; here *n* is the number of variables and 0 corresponds to the central point. The experimental levels for these variables were preferred from our preliminary work, which indicated that an optimum could be built within the level of processes parameters calculated. The levels of factors used for experimental design are given in Table 4. The actual level of each factor was calculated by the following equation [18]:

(2) $$\begin{array}{c} \text{Coded}\;\;\text{value}=\frac{\text{actual}\;\;\text{level}-\left(\text{high}\;\;\text{level}+\text{low}\;\;\text{level}\right)/2}{\left(\text{high}\;\;\text{level}-\text{low}\;\;\text{level}\right)/2}. \end{array}$$

The experimental design scheme is given in Table 5. Rifamycin B yield was analyzed by using a second-order polynomial equation and the data were fitted into the equation by multiple regression procedure. The model equation for analysis is given below:

(3) Y=β0+β1X1+β2X2+β3X3+β4X4+β11X12 +β22X22+β33X32+β44X42+β12X1X2 +β24X2X4+β13X1X3+β14X1X4 +β23X2X3+β24X2X4+β34X3X4,

where *Y* is the predicted response, *X*_1_,…, *X*_4_ are the levels of the factors, *β*_1_,…, *β*_4_ are linear coefficients, *β*_11_,…, *β*_44_ are quadratic coefficients, and *β*_12_,…, *β*_34_ are the interactive coefficients with *β*_0_ is a scaling constant. Analysis of variance (ANOVA), regression analysis was done. The surface and contour plots were drawn by using Stat-Ease Design Expert statistical software package (trial version7).

### 2.10. Data Analysis

The quality of the fit of the polynomial model equation was uttered by the coefficient of determination *R* ^2^, and its statistical significance was checkered by *F*-test. The significance level was given as values of Prob > *F* less than 0.1. A differential calculation was then engaged for predicting the optimum point.

## 3. Results and Discussion

### 3.1. Screening of Carbon and Nitrogen Sources, Barbital, pH, and Moisture Content for Production of Rifamycin B

In the introductory step of optimization, the preferred nutrients and physical parameters were applied to sunflower oil cake separately. The complementary nutrients and physical parameters really advance concentration of rifamycin B of* Nocardia mediterranei* MTCC 14.

**Figure 1:**
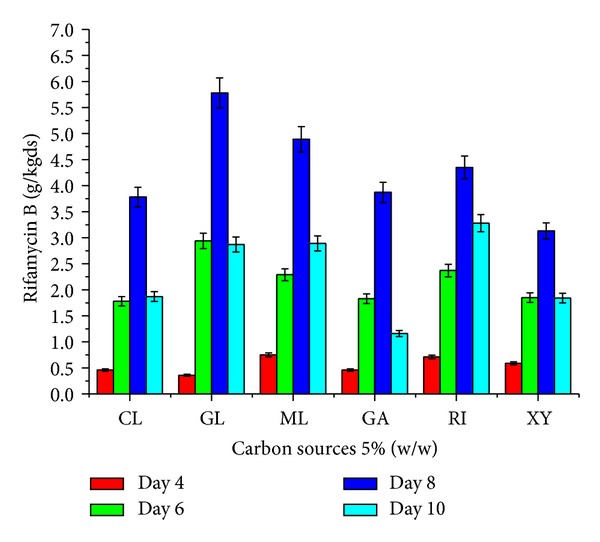
Effect of various carbon sources (5% w/w) on rifamycin B production by* Nocardia mediterranei* MTCC 14 using sunflower oil cake under solid-state fermentation. Cl: control, GL: glucose, Ml: maltose, Ga: galactose, RI: ribose, and XY: xylose.

**Figure 2:**
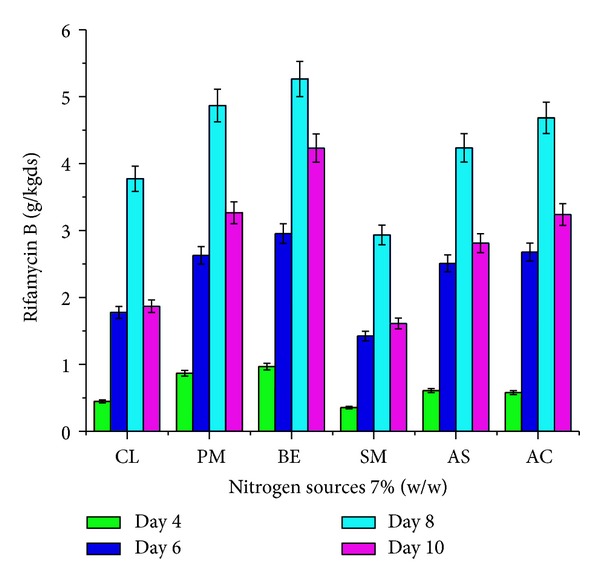
Effect of various nitrogen sources (7% w/w) on rifamycin B production by* Nocardia mediterranei* MTCC 14 using sunflower oil cake under solid-state fermentation. Cl: control, PM: peanut meal, Be: beef extract, SM: soybean meal, AS: ammonium sulphate, and AC: ammonium chloride.

**Figure 3:**
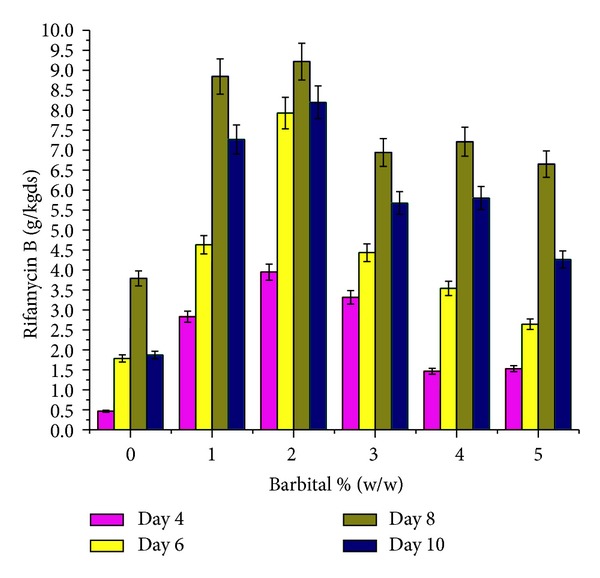
Effect of various concentrations of barbital (% w/w) on rifamycin B production by* Nocardia mediterranei* MTCC 14 using sunflower oil cake under solid-state fermentation.

**Figure 4:**
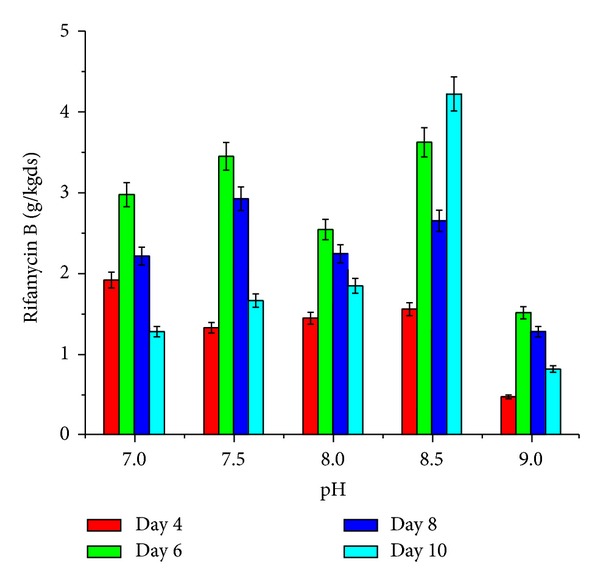
Effect of various levels of pH on rifamycin B production by* Nocardia mediterranei* MTCC 14 using sunflower oil cake under solid-state fermentation.

**Figure 5:**
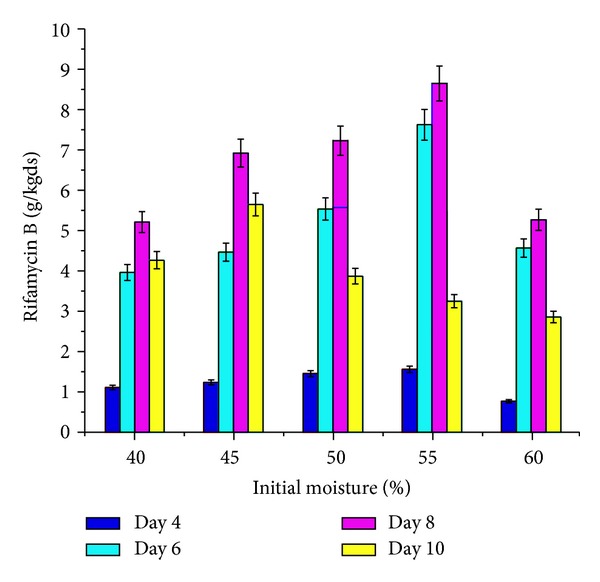
Effect of various levels of moisture (%) on rifamycin B production by* Nocardia mediterranei* MTCC 14 using sunflower oil cake under solid-state fermentation.

The effect of supplementation with various carbon and nitrogen sources and barbital and application of physical parameters such as pH and moisture content of the medium on rifamycin B production by* Nocardia mediterranei* MTCC 14 is shown in Figures 1–5. Carbon and nitrogen sources, in the basal medium, were at a level of 5% (w/w) and 7% (w/w), barbital of 1%, 2%, 3%, 4%, and 5%, pH of 7.0, 7.5, 8.0, 8.5, and 9.0, and moisture content of 40%, 45%, 50%, 55%, and 60%.

The effects of supplementation with carbon sources were studied at a level of 5% w/w and were generally found to have a good effect on the production of rifamycin B (Figure 1). Glucose, maltose, ribose, and galactose supplementation showed the highest production of rifamycin B at 5.78, 4.89, 4.35, and 3.87 g/kgds dry sunflower oil cake at the day eight of incubation. These data further indicate that though the sunflower oil cake material provides carbon source required for* Nocardia mediterranei* MTCC 14 growth and subsequent rifamycin B production, it was observed to be insufficient medium for optimum production. Such improved metabolite production values were also reported for* Amycolatopsis* sp. RSP 3 with supplementation of external carbon sources under SSF [6].

Rifamycin B production in different microbial strains is reported to be supported by the presence of various organic nitrogen sources such as beef extract [6], soybean meal [19], peanut meal [20], ammonium sulphate [6], and ammonium chloride [6] during solid-state fermentation. Hence, the effect of different nitrogen sources on rifamycin B production by* Nocardia mediterranei* MTCC 14 was studied at optimized SSF environment. The effect of the supplementation of nitrogen sources was studied by adding various nitrogen sources at a concentration of 7% w/w. Results indicated that they almost completely stimulated the production of rifamycin B at the day eight of incubation. Soybean meal did not affect rifamycin B yield (Figure 2) compared to control (CL). Almost all the nitrogen sources tested increased the rifamycin B production at the day eight of incubation.

Rifamycin B production is affected by the appearance of barbital in the fermentation medium [21, 22]. It is reported that barbital negatively regulates the electron transport system and increases the accessibility of oxygen to other cellular activities which in turn improves rifamycin production [23]. Supplementation with various concentrations of barbital gave a significantly higher rifamycin B yield at day 8 of incubation (Figure 3).

The influence of wide ranges of pH from 7 to 9 [6] on the production of rifamycin B from corn husk has been reported. Results revealed (Figure 4) that pH 8.5 was optimum for the maximum production of rifamycin B from sun flower oil cake in solid-state conditions. Krishna et al. [24] also reported similar observations. Wide range of initial pH of the medium during the upstream bioprocess makes the end product either acidic or alkaline, which tends to have varied applications [25]. However, the influence of pH on the production of rifamycin B from sun flower oil cake was significant when compared to submerged conditions.

Different amounts of water were added to dry sunflower oil cake, and the effect on rifamycin B production is shown in Figure 5, where the maximum production of rifamycin B was 8.65 g/kgds dry sunflower oil cake at 55% moisture content. Increasing the moisture content beyond 60% caused a decrease in the production of rifamycin B, which may be due to poor gas exchange as a result of water batting the pore spaces between the solid medium particles. Similarly, Mahalaxmi et al. [6] used 57% moisture content for the production of rifamycin B from corn husk.

Based on the above experiments, glucose, maltose, ribose, galactose, beef extract, peanut meal, ammonium chloride, ammonium sulphate, barbital, pH, and moisture content were selected for statistical optimization.

### 3.2. Screening of Production Parameters Using Plackett-Burman Design Test

**Figure 6:**
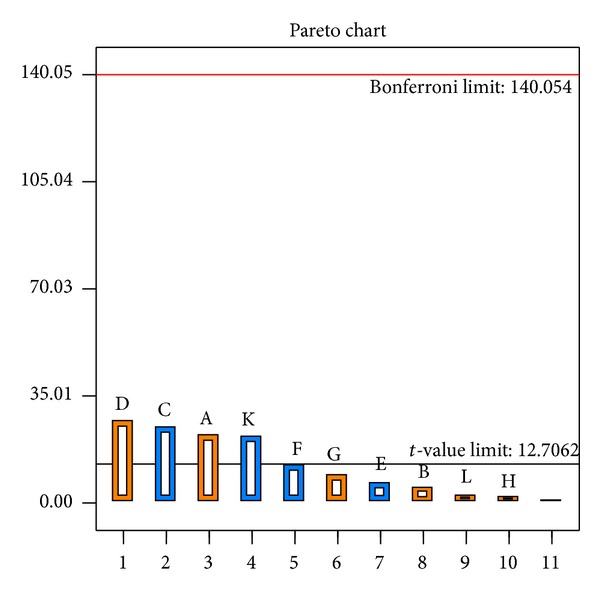
*X*1: Rank of process variables; *X*2: *t*-value of effect. Pareto chart of independent variables (glucose (A; *X*1), maltose (B; *X*2), ribose (C; *X*3), galactose (D; *X*4), beef extract (E; *X*5), peanut meal (F; *X*6), ammonium chloride (G; *X*7), ammonium sulphate (H; *X*8), barbital (I; *X*9), pH (j; *X*10), and moisture content (K; *X*11)) for rifamycin B production by* Nocardia mediterranei* MTCC 14 using sunflower oil cake under solid-state fermentation.

A total of eleven variables were analyzed with regard to their effects on rifamycin B yield using a Plackett-Burman design (Table 1). The design matrix selected for screening of significant variables for rifamycin B production and the corresponding responses are shown in Table 2. The adequacy of the model was calculated, and the variables evidencing statistically significant effects were screened via regression analysis (Table 3). Among the eleven production parameters (glucose, maltose, ribose, galactose, beef extract, peanut meal, ammonium chloride, ammonium sulphate, barbital, pH, and moisture content) studied, four parameters (galactose, ribose, glucose, and pH) were found to have significant influence on rifamycin B production as evidenced by their *P* values (<0.05, significant at 5% level) obtained from regression analysis. We used the Pareto chart in order to calculate the amplitude and worth of an independent variable and its interaction with the dependent variable (Figure 6). The chart displays the actual value of the effects and draws a reference line on the chart. The length of each bar in the chart indicates the standardized effect of that factor on the response. Any effect that extends past this reference line is possibly significant for that certain process [26]. The fact that the bars for maltose, beef extract, peanut meal, ammonium chloride, ammonium sulphate, barbital, and moisture content remained inward the reference line as shown in Figure 6 denotes that these terms contributed to the least prediction of the rifamycin B production. The coefficient of determination (*R* ^2^) of the model was found to be 0.9996 which indicates that the model can explain up to 99.96% variation of the data. Rifamycin B yield obtained from Plackett-Burman design experiments showed wide variation (0.2–1.9%), which indicated that further optimization is necessary to get a maximum response.

### 3.3. Effects of Galactose, Ribose, Glucose, and pH on Rifamycin B Production

Response surface methodology using central composite design was practical to optimize the levels of significant production parameters resulting from Plackett-Burman design experiments. Thirty experiments were carried out from the design and the experimental values are given in Table 5. All the experiments were carried out in duplicate; the mean value of rifamycin B yield was taken for statistical analysis. By applying multiple regression analysis on the experimental data, the following second-order polynomial equation was developed:

(4) Rifamycin  B  (g/kgds) =+2306.89958−97.84583X4−244.48417X3  −14.31583X1−384.39167X10+6.26500X4X3  +1.50000X4X1−1.21000X4X10−2.11000X3X1  +20.15000X3X10−0.98000X1X10+4.42750X42  +9.07750X32+0.89750X12+22.55000X102.

**Figure 7:**
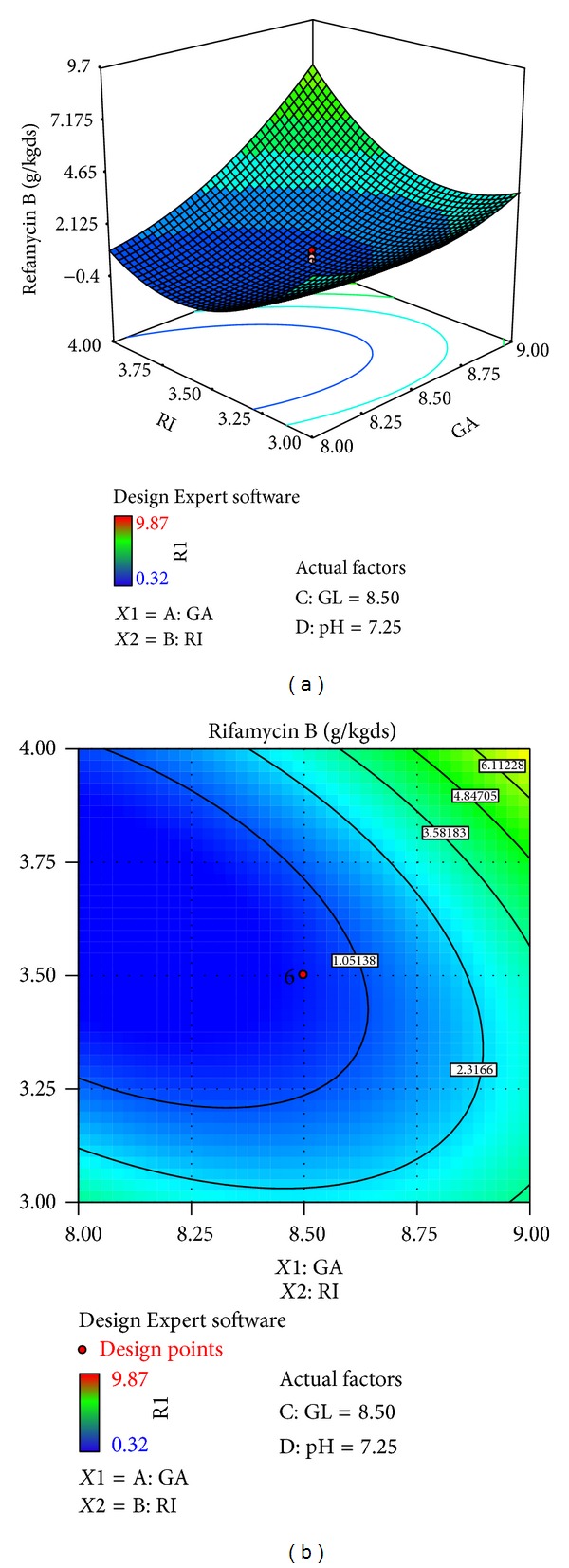
Surface and contour plot for rifamycin B production at varying concentrations of *X*4, galactose (GA), and *X*3, ribose (RI).

**Figure 8:**
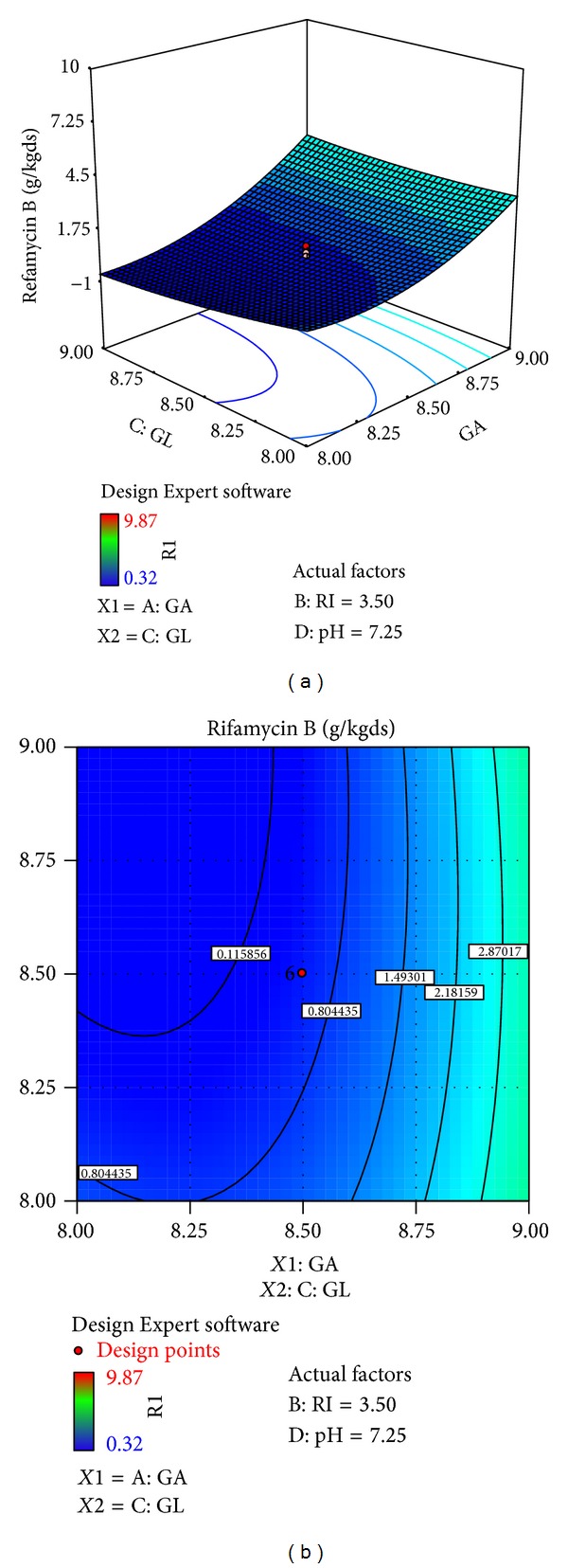
Surface and contour plot for rifamycin B production at varying concentrations of *X*4, galactose (GA), and *X*1, glucose (GL).

**Figure 9:**
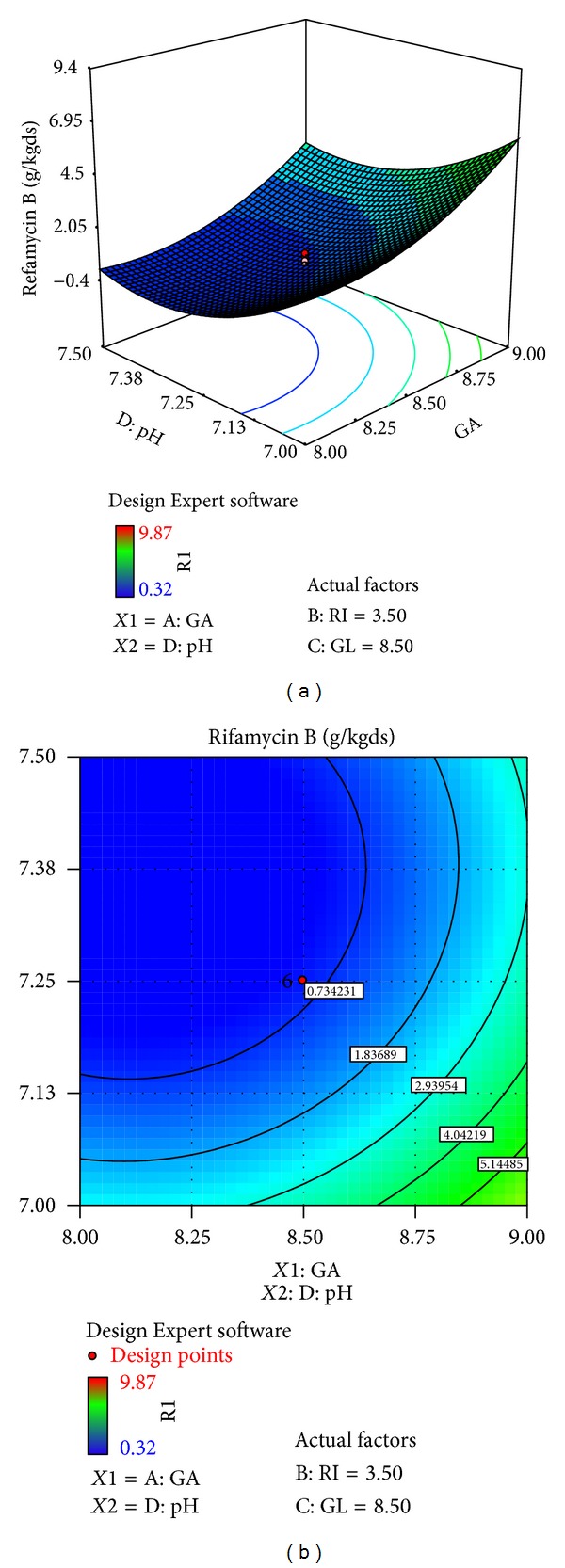
Surface and contour plot for rifamycin B production at varying levels of *X*1, galactose (GA), and *X*10, pH.

**Figure 10:**
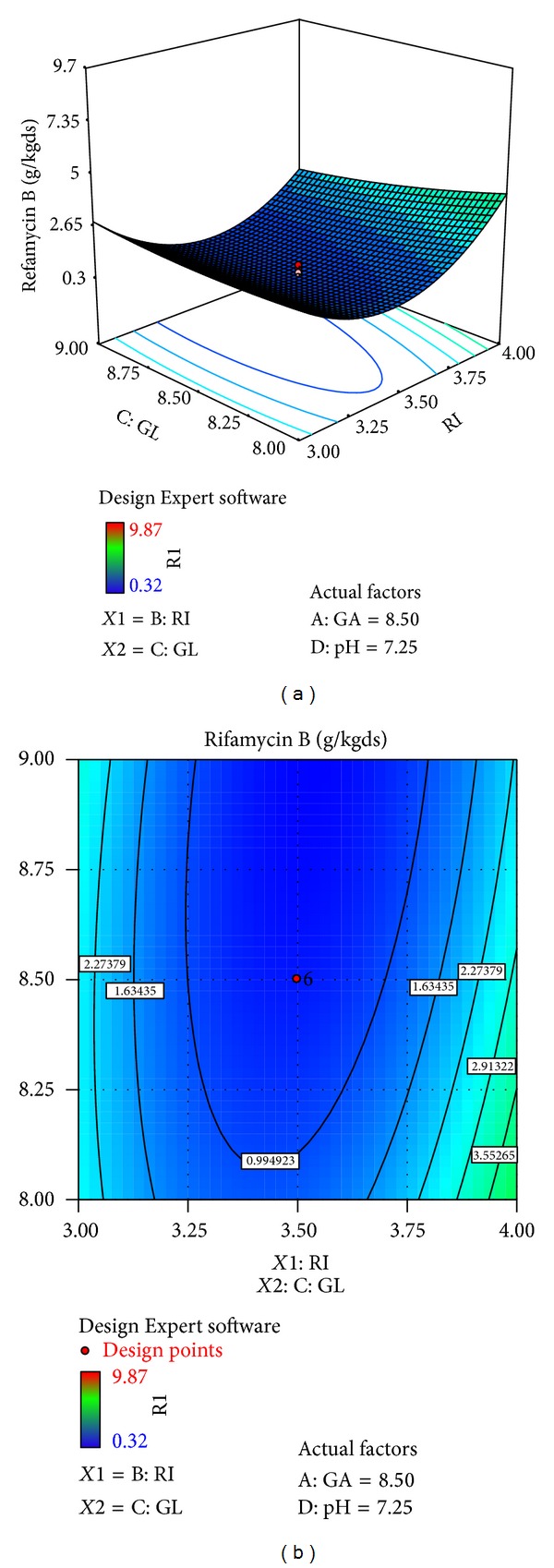
Surface and contour plot for rifamycin B production at varying concentrations of *X*3, ribose (RI), and *X*1, glucose (GL).

**Figure 11:**
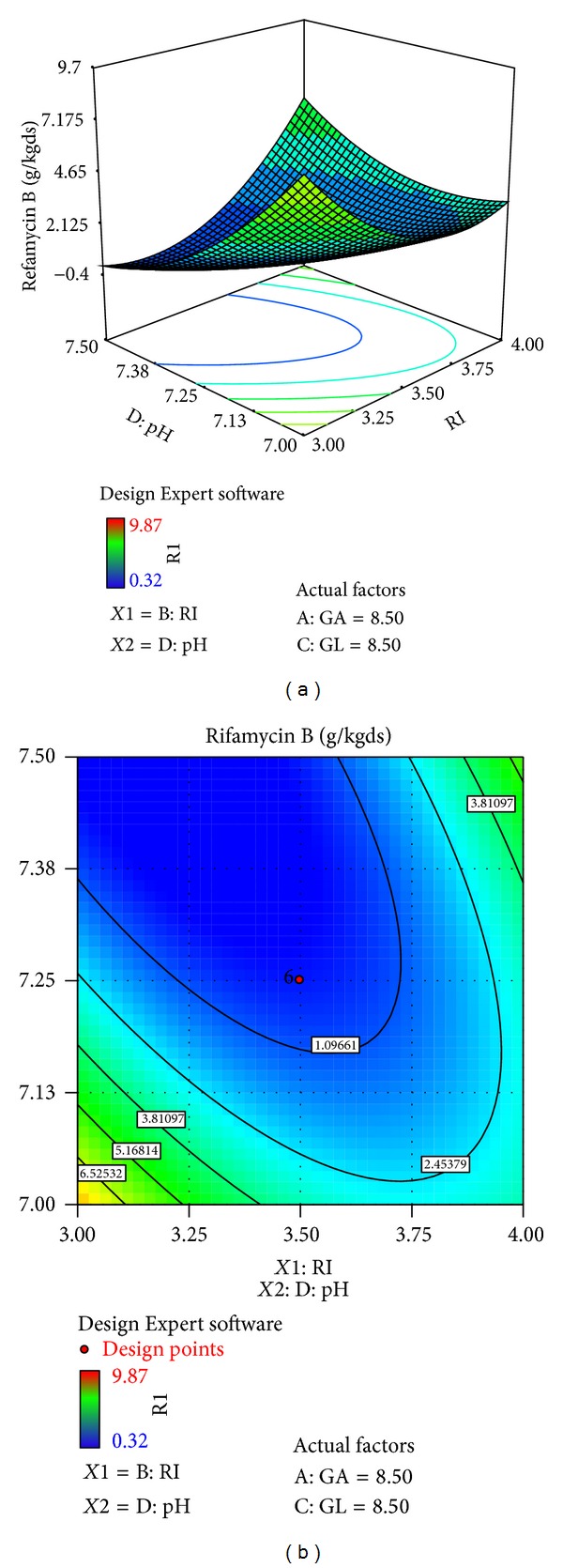
Surface and contour plot for rifamycin B production at varying levels of *X*3, ribose (RI), and *X*10, pH.

**Figure 12:**
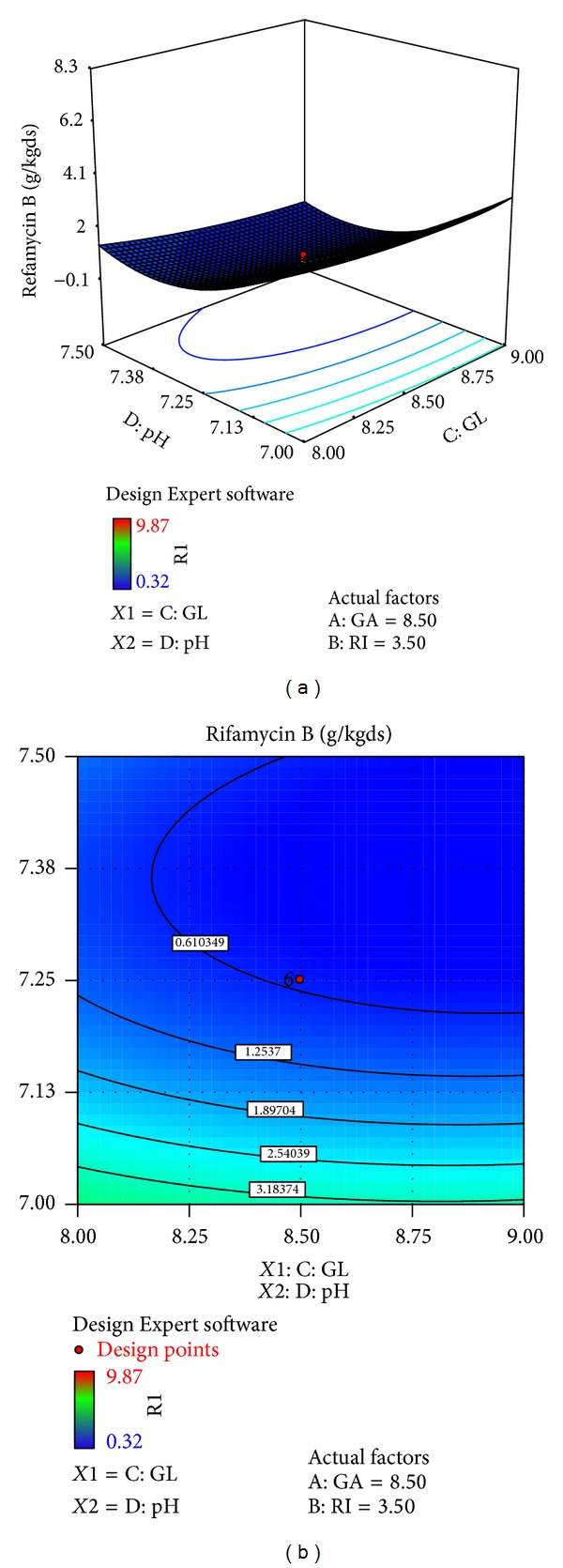
Surface and contour plot for rifamycin B production at varying levels of *X*1, glucose (GL), and *X*10, pH.

The effects of galactose (*X*4), ribose (*X*3), glucose (*X*1), and pH (*X*10) on rifamycin B production are reported in Table 7. Response surfaces for rifamycin B yield are shown in Figures 7–12, which give the surface and contour plots for the effect of galactose, ribose, glucose, and pH on the rifamycin B yield. Regression analysis of the experimental data (Table 7) showed that galactose and ribose had significant positive linear effects on rifamycin B yield, while glucose and pH had negative linear effect on rifamycin B yield. This was clear from the low *P* value obtained from the regression analysis. Among the four processes parameters, galactose was found to have the highest impact on rifamycin B yield as given by the highest linear coefficient (1.66) followed by ribose (0.23), while glucose (−0.40) and pH (−1.38) have negative linear effect. These production parameters also showed significant positive quadratic effects on rifamycin B yield indicating that rifamycin B production increased as the level of these factors increased and decreased as the level of these processes parameters decreased below certain values. Table also indicates that the interaction between ribose and pH and between galactose and ribose has significant effect on rifamycin B production and all other interactive variables are insignificant. Hence, only the term indicating interaction between ribose and pH and between galactose and ribose was included in the model regression equation (4).

Figures 7–12 show the surface and contour plots of rifamycin B produced for each pair of production parameters by keeping the other two production parameters constant at its central level. The effect of galactose and ribose on the production of rifamycin B is shown in Figure 7. The maximum rifamycin B (8.34 g/kgds) was obtained at galactose 8.5% w/w and ribose 3.25% w/w. Further increase in concentration of galactose and ribose leads to increase in production of rifamycin B.  Figure 8 indicates that the maximum rifamycin B was produced (5.57 g/kgds) when galactose and glucose were 8% w/w and 9% w/w; further increase in the concentration of galactose leads to increase in production of rifamycin B and further decrease in the concentration of glucose leads to increase in production of rifamycin B. The surface and contour plot of Figure 9 indicates that the maximum rifamycin B (4.80 g/kgds) production occurred at the galactose of 9% w/w and pH of 7. The production of rifamycin B increases with increase in concentration of galactose up to 9% w/w and decrease in pH up to 7 and further increase in the concentration of galactose and further decrease in pH lead to increase in the production of rifamycin B.  Figure 10 indicates that the maximum rifamycin B is produced at the concentrations of ribose above 4% w/w and below 3% w/w and glucose above 9% w/w and below 8% w/w. Maximum rifamycin B was produced when concentration of ribose was above 4% w/w and below 3% w/w and pH was 7 (Figure 11). Further decrease in pH leads to acceleration of rifamycin B production.  Figure 12 surface and contour plot shows that maximum rifamycin B was produced at concentration of glucose of 9% w/w and pH was 7.

### 3.4. Statistical Analysis

Analysis of variance for the rifamycin B produced from* Nocardia mediterranei* MTCC 14 obtained from this design was given in Table 6. ANOVA gives the value of the model and can account for whether this model adequately fits the variation observed in rifamycin B produced with the designed production level. If the *F*-test for the model is significant at the 5% level (*P* < 0.05), then the model is fit and can satisfactorily explain the variation ascertained. If the *F*-test for lack of fit is significant (*P* < 0.05), then a more complex model is essential to adapt the data. The high *F*-value and nonsignificant lack of fit indicated that the quadratic model was highly significant, as reported in [27, 28]. The *P* value for lack of fit (0.0006) reveals that the experimental data obtained fit well with the model and explains the effect of galactose, ribose, glucose, and pH on rifamycin B produced from the* Nocardia mediterranei* MTCC 14. The nearer the value of *R* ^2^ (coefficient of determination) to 1, the more correct the correlation between the observed and predicted values. Here the value of *R* ^2^ (0.9547) indicates that the model can explain up to 95.47% variation of rifamycin B yield obtained. A coefficient of variance (CV) value of 26.34 reveals the high precision and reliability of the experimental data. Adequate precision greater than 4.0 is desirable, and the ratio was found to be 12.510 (>4.0), which indicates that the derived model is significant for the rifamycin B production. The predicted residual sum of squares (PRESS) value was 122.79, which showed how the derived model fits with each point in the given design [29].

### 3.5. Adequacy of the Model

**Figure 13:**
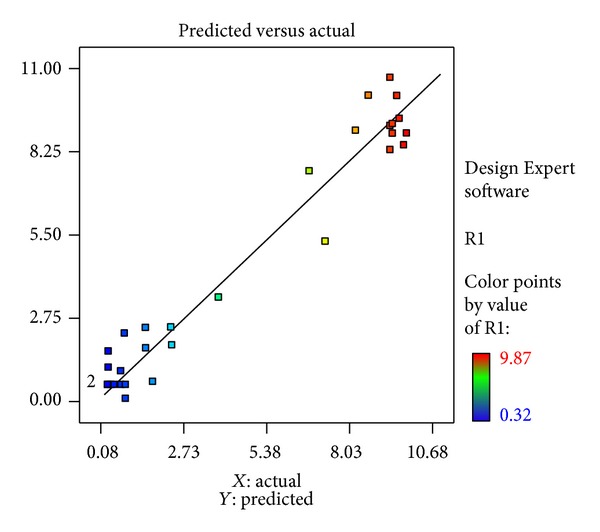
Plot between actual and predicted yield of rifamycin B.

Model adequacy scrutiny was used, in order to find whether the derived model would give sufficient approximation values to the actual values [30]. Raw residuals are elements of variation for the given data, which cannot be explained by the model, representing the deviations between the experimental and predicted values. The predicted values from the model were quite close to the experimental values, and the data of all predicted and experimental response values lie in a straight line (Figure 13) indicating that the derived model was able to well predict the correlation between the process variables on the response.

### 3.6. Validation of the Model

The experimental data were fitted into the model (4) and the optimum values were found to be as follows: galactose 8% w/w, ribose 3% w/w, glucose 9% w/w, and pH 7.0. Optimum levels of production parameters for production of rifamycin B from* Nocardia mediterranei* MTCC 14 was 9.87 g/kgds, which is very close to the predicted value of 10.35 g/kgds.

## 4. Conclusion

Response surface methodology was well used to optimize the production parameters for production of rifamycin B from* Nocardia mediterranei* MTCC 14. To optimize various processes parameters for production of rifamycin B from* Nocardia mediterranei* MTCC 14 eleven processes parameters (glucose, maltose, ribose, galactose, beef extract, peanut meal, ammonium chloride, ammonium sulphate, barbital, pH, and moisture content) were tried by using Plackett-Burman design test and the four processes parameters galactose, ribose, glucose, and pH showed significant effects on production of rifamycin B. The actuality of interactions between the processes parameters was calculated and the interaction between ribose and pH and between galactose and ribose showed significant effects on production of rifamycin B. The production parameters were optimized by applying central composite design and the parameters for the highest production of rifamycin B from* Nocardia mediterranei* MTCC 14 were found to be galactose (8% w/w), ribose (3% w/w), glucose (9% w/w), and pH (7.0). The maximum rifamycin B yield from* Nocardia mediterranei* MTCC 14 was 9.87 g/kgds dry sunflower oil cake. The second-order polynomial model developed was found to be acceptable in describing the experimental data. This is the first report of the optimization of production of rifamycin B from* Nocardia mediterranei* MTCC 14 using response surface methodology.

## Figures and Tables

**Table 1:** Level of the production parameters for production of rifamycin B from *Nocardia mediterranei* MTCC 14 by using Plackett-Burman design test.

Processes parameters code	Medium composition and conditions	High level (+)	Low level (−)
*X*1	Glucose % (w/w)	7	5
*X*2	Maltose % (w/w)	7	5
*X*3	Ribose % (w/w)	7	5
*X*4	Galactose % (w/w)	7	5
*X*5	Beef extract % (w/w)	9	7
*X*6	Peanut meal % (w/w)	9	7
*X*7	Ammonium chloride % (w/w)	9	7
*X*8	Ammonium sulphate % (w/w)	9	7
*X*9	Barbital % (w/w)	3	2
*X*10	pH	9	7
*X*11	Moisture content %	60	50

**Table 2:** Yield of rifamycin B from *Nocardia mediterranei* MTCC 14 using the different levels of production variables of Plackett-Burman design test.

Std. run order	*X*1	*X*2	*X*3	*X*4	*X*5	*X*6	*X*7	*X*8	*X*9	*X*10	*X*11	Yield g/kgds
2	−1	1	1	−1	1	1	1	−1	−1	−1	1	5.21
9	1	1	1	−1	−1	−1	1	−1	1	1	−1	6.38
1	1	1	−1	1	1	1	−1	−1	−1	1	−1	7.84
5	−1	−1	1	−1	1	1	−1	1	1	1	−1	2.89
10	−1	1	1	1	−1	−1	−1	1	−1	1	1	6.32
11	1	−1	1	1	1	−1	−1	−1	1	−1	1	8.35
6	−1	−1	−1	1	−1	1	1	−1	1	1	1	7.32
12	−1	−1	−1	−1	−1	−1	−1	−1	−1	−1	−1	6.97
8	1	1	−1	−1	−1	1	−1	1	1	−1	1	8.31
7	1	−1	−1	−1	1	−1	1	1	−1	1	1	7.47
4	−1	1	−1	1	1	−1	1	1	1	−1	−1	9.43
3	1	−1	1	1	−1	1	1	1	−1	−1	−1	8.48

Eleven variables, *X*1, glucose, *X*2, maltose, *X*3, ribose, *X*4, galactose, *X*5, beef extract, *X*6, peanut meal, *X*7, ammonium chloride, *X*8, ammonium sulphate, *X*9, barbital, *X*10, pH, and *X*11, moisture content, were screened by conducting 12 experiments.

**Table 3:** ANOVA of Plackett-Burman design test data for the significant production parameters.

Source	SS	df	MS	*F* value	*P* value
Model	33.52	11	3.35	264.42	0.0478
Glucose	6.29	1	6.29	496.49	0.0286
Maltose	0.34	1	0.34	26.56	0.1220
Ribose	7.86	1	7.86	619.88	0.0256
Galactose	9.21	1	9.21	726.23	0.0236
Beef extract	0.56	1	0.56	44.10	0.0951
Peanut meal	1.98	1	1.98	155.93	0.0509
Ammonium chloride	1.09	1	1.09	85.68	0.0685
Ammonium sulphate	0.057	1	0.057	4.53	0.2796
Barbital	0.013	1	0.013	3.78	0.9522
pH	6.06	1	6.06	478.38	0.0291
Moisture content	0.082	1	0.082	6.44	0.2389

*R* ^2^ = 0.9996					

Galactose, ribose, glucose, and pH were significant (*P* < 0.05). SS: sum of square, MS: mean of square, df: degree of freedom, *F* value: Fisher's value, and *P*: probability.

**Table 4:** Production variables and their coded and actual values used for optimization of rifamycin B production from *mediterranei* MTCC 14 by using central composite design.

Production variable	Coded level					
	Symbol	−2	−1	0	1	2
Galactose % (w/w)	*X*4	7.50	8.0	8.50	9.0	9.50
Ribose % (w/w)	*X*3	2.50	3.0	3.50	4.0	4.50
Glucose % (w/w)	*X*1	7.50	8.0	8.50	9.0	9.50
pH % (w/w)	*X*10	6.75	7.0	7.25	7.5	7.75

**Table 5:** Experimental design and results using central composite design.

Std. run order	Production parameters levels				Yield g/kgds
	*X*4	*X*3	*X*1	*X*10	
7	8.00	4.00	9.00	7.00	0.89
30	8.50	3.50	8.50	7.25	0.32
11	8.00	4.00	8.00	7.50	7.27
15	8.00	4.00	9.00	7.50	1.53
18	9.50	3.50	8.50	7.25	9.34
2	9.00	3.00	8.00	7.00	9.42
29	8.50	3.50	8.50	7.25	0.53
28	8.50	3.50	8.50	7.25	0.89
27	8.50	3.50	8.50	7.25	0.43
26	8.50	3.50	8.50	7.25	0.75
21	8.50	3.50	7.50	7.25	0.86
8	9.00	4.00	9.00	7.00	6.76
9	8.00	3.00	8.00	7.50	2.37
20	8.50	4.50	8.50	7.25	9.56
13	8.00	3.00	9.00	7.50	0.34
14	9.00	3.00	9.00	7.50	1.54
4	9.00	4.00	8.00	7.00	9.78
6	9.00	3.00	9.00	7.00	8.65
1	8.00	3.00	8.00	7.00	9.34
12	9.00	4.00	8.00	7.50	9.34
16	9.00	4.00	9.00	7.50	9.64
22	8.50	3.50	9.50	7.25	1.76
25	8.50	3.50	8.50	7.25	0.32
23	8.50	3.50	8.50	6.75	8.24
24	8.50	3.50	8.50	7.75	3.86
3	8.00	4.00	8.00	7.00	2.34
19	8.50	2.50	8.50	7.25	9.42
10	9.00	3.00	8.00	7.50	0.74
5	8.00	3.00	9.00	7.00	9.87
17	7.50	3.50	8.50	7.25	0.34

*X*4: galactose, *X*3: ribose, *X*1: glucose, and *X*10: pH.

**Table 6:** ANOVA for the fitted quadratic polynomial model of rifamycin B production from *mediterranei* MTCC 14 by using central composite design.

Source	SS	DF	MS	*F* value	*P* value	Significance
Model	453.92	14	32.42	22.59	<0.0001	Significant
*X*4	66.40	1	66.40	46.27	<0.0001	Significant
*X*3	1.29	1	1.29	0.90	0.3584	Not significant
*X*1	3.82	1	3.82	2.66	0.1234	Not significant
*X*10	45.49	1	45.49	31.70	<0.0001	Significant
*X*4*X*3	39.25	1	39.25	27.35	0.0001	Significant
*X*4*X*1	2.25	1	2.25	1.57	0.2297	Not significant
*X*4*X*10	0.37	1	0.37	0.26	0.6209	Not significant
*X*3*X*1	4.45	1	4.45	3.10	0.0985	Not significant
*X*3*X*10	101.51	1	101.51	70.74	<0.0001	Significant
*X*1*X*10	0.24	1	0.24	0.17	0.6833	Not significant
*X*4^2^	33.60	1	33.60	23.42	0.0002	Significant
*X*3^2^	141.26	1	141.26	98.44	<0.0001	Significant
*X*1^2^	1.38	1	1.38	0.96	0.3422	Not significant
*X*10^2^	54.48	1	54.48	37.97	<0.0001	Significant
Residual	21.52	15	1.43			
Lack of fit	21.25	10	2.12	38.55	0.0004	Not significant
Pure error	0.28	5	0.055			
Cor. total	475.45	29				

Std. dev.: 1.20, *R* ^2^: 0.9547. Mean: 4.55, adjusted *R* ^2^: 0.9125. Coefficient of variation (CV) %: 26.34, predicted *R* ^2^: 0.7118. Predicted residual sum of squares (PRESS): 122.79, adequate precision: 12.510.						

SS: sum of square, MS: mean of square, and df: degree of freedom.

**Table 7:** Coefficients of the multivariate process models for the rifamycin B production from *mediterranei* MTCC.

Factor	Coefficient of estimate	Standard error
Intercept	0.54	0.49
*X*4	1.66	0.24
*X*3	0.23	0.24
*X*1	−0.40	0.24
*X*10	−1.38	0.24
*X*4*X*3	1.57	0.30
*X*4*X*1	0.37	0.30
*X*4*X*10	−0.15	0.30
*X*3*X*1	−0.53	0.30
*X*3*X*10	2.52	0.30
*X*1*X*10	−0.12	0.30
*X*4^2^	1.11	0.23
*X*3^2^	2.27	0.23
*X*1^2^	0.22	0.23
*X*10^2^	1.41	0.23
